# Recorded Adoption of SGLT2 Inhibitors from 2022 to 2025 Among Adults with an EHR-Defined HFrEF Phenotype at a Jordanian Public Hospital

**DOI:** 10.3390/jcm15197569

**Published:** 2026-09-29

**Authors:** Tala Bassam Al-Bawalsah, Anas Abed, Alhareth Ahmad, Sireen Abdul Rahim Shilbayeh

**Affiliations:** 1 Department of Biopharmaceutics and Clinical Pharmacy, Faculty of Pharmacy, Al-Ahliyya Amman University, Amman 19328, Jordan; 202317230@ammanu.edu.jo (T.B.A.-B.); a.alsad@ammanu.edu.jo (A.A.); 2 Department of Pharmacy Practice, Princess Nourah bint Abdulrahman University, P.O. Box 84428, Riyadh 11671, Saudi Arabia; ssabdulrahim@pnu.edu.sa

**Keywords:** heart failure, HFrEF, SGLT2 inhibitors, dapagliflozin, empagliflozin, electronic health records, guideline-directed medical therapy, Jordan

## Abstract

**Background/Objectives:** Sodium-glucose cotransporter-2 inhibitors (SGLT2i) are foundational therapy for heart failure with reduced ejection fraction (HFrEF), irrespective of diabetes. We evaluated recorded SGLT2i adoption and diabetes-related differences among adults with an electronic health record (EHR)-defined HFrEF phenotype at a Jordanian public hospital. **Methods:** This retrospective Hakeem cohort included 1750 adults. The frozen HFrEF phenotype was internally validated in an independent non-overlapping sample, and SGLT2i ascertainment was verified by complete-cohort medication review. Comparable annual analyses covered 2022–2025, when medication-source completeness exceeded 90%. Secondary analyses examined first recorded use after a 180-day lookback, concurrent four-class guideline-directed medical therapy (GDMT) in 2025, measured-eGFR threshold cohorts, and prespecified Firth regression among patients with type 2 diabetes (T2DM). Additional sensitivity analyses included a stable four-year cohort, a time-to-first-record analysis with explicit censoring, expanded model diagnostics with a time-to-event sensitivity analysis, and a strict directly documented-coverage GDMT analysis. **Results:** Sixty-four patients had verified recorded SGLT2i use (3.66%; 95% CI 2.83–4.65). Use was 11.41% (64/561) with T2DM and 0% (0/1189; exact 95% CI 0–0.31) without diabetes. Annual prevalence increased from 0.15% in 2022 to 5.13% in 2025 (absolute increase 4.98 percentage points), while remaining 0% without diabetes. In the stable four-year cohort sensitivity analysis, prevalence similarly increased from 0.21% to 5.34%. In the verified 12-month fixed-horizon cohort, 27/1268 patients (2.13%) had a first recorded use within one year; the 322-day median was conditional on the 50 patients who eventually had a first post-index record and was not interpreted as a cohort-wide waiting time. Only 24/936 patients (2.56%) had all four foundational classes concurrently active in the primary 2025 analysis. The inverse nondialysis-CKD estimate was treated as exploratory because only four treated patients had nondialysis CKD. **Conclusions:** Recorded adoption improved but remained low and strongly concentrated among patients with T2DM. Multicenter linkage of prescribing, eligibility, dispensing, persistence, and reimbursement data is needed to identify implementation mechanisms.

## 1. Introduction

Heart failure (HF) remains a major cause of hospitalization, disability, and premature mortality. Contemporary management of HF with reduced ejection fraction (HFrEF) centers on rapid implementation of complementary disease-modifying therapies. The 2022 American and 2021/2023 European recommendations established four foundational treatment classes: renin-angiotensin system inhibition, preferably with an angiotensin receptor-neprilysin inhibitor where appropriate; an evidence-based beta-blocker; a mineralocorticoid receptor antagonist; and a sodium-glucose cotransporter-2 inhibitor (SGLT2i) [1,2,3,4]. The 2026 European Society of Cardiology guideline has since updated HF classification and terminology while retaining emphasis on timely evidence-based treatment [5].

DAPA-HF and EMPEROR-Reduced demonstrated that dapagliflozin and empagliflozin reduce worsening HF events in HFrEF, with treatment effects that are consistent in patients with and without type 2 diabetes mellitus (T2DM) [6,7,8,9]. SGLT2i initiation is comparatively straightforward because the HF dose is fixed and does not require titration, although kidney function, volume status, acute illness, ketoacidosis risk, and dialysis require clinical assessment [10,11,12]. These characteristics make SGLT2i particularly suitable for early integration into multidisciplinary HF pathways [13].

Translation into routine practice remains incomplete and variable. In the US Get With The Guidelines-HF registry, 20.2% of 49,399 eligible patients hospitalized with HFrEF received an SGLT2i at discharge [14]. A 2026 multicenter Saudi study reported substantially higher use among patients receiving longitudinal tertiary HF care, but also identified lower use with advanced kidney disease [15]. These estimates cannot be directly compared because populations, observation periods, eligibility criteria, and medication sources differ; collectively, however, they show that adoption is shaped by both clinical and health-system context.

Jordanian evidence provides two essential points of comparison. The Jordan Heart Failure Registry reported SGLT2i use in 9.0% of 2151 patients across 21 centers during 2021–2023, but SGLT2i-specific longitudinal adoption and diabetes-stratified patient-level determinants were not its primary focus [16]. A subsequent explanatory mixed-methods study involving treating and insurance physicians identified misclassification of SGLT2i as diabetes-only medicines, prior authorization, limited confidence outside cardiology, and delayed approvals as potential implementation mechanisms [17]. That stakeholder study did not include a longitudinal patient-level prescription cohort.

The present study was designed as a complementary patient-level analysis rather than a repetition of those investigations. Its objectives were to (1) quantify verified recorded SGLT2i use among adults having a reproducible EHR-based phenotype compatible with HFrEF; (2) characterize annual adoption during the comparable-data period from 2022 to 2025; (3) quantify differences by diabetes status; (4) examine first recorded use among patients with complete baseline medication observability; (5) describe concurrent foundational GDMT at a standardized 2025 assessment; and (6) test robustness across strict-phenotype, observable-medication, measured-eGFR threshold, fixed-horizon, and broad-HF cohorts. We hypothesized that recorded use would increase over time but remain concentrated among patients with T2DM.

## 2. Materials and Methods

### 2.1. Design, Setting, and Reporting

We conducted a retrospective cohort study using Hakeem EHR data from Al-Bashir Hospital, a large public tertiary and referral hospital in Amman, Jordan. Source records extended from 1 January 2020 through 31 December 2025. Because linked medication-source completeness was below 50% in 2020–2021 and exceeded 90% from 2022 onward, the prespecified comparable-period analysis was restricted to 1 January 2022 through 31 December 2025. Records from 2020–2021 were used only for baseline lookback, phenotype development, and a transparent data-coverage audit; they were not interpreted as clinical adoption estimates. Hakeem is deployed across multiple Jordanian facilities, but the present analysis used Al-Bashir Hospital data only and was not considered nationally representative. Reporting followed the RECORD extension of STROBE [18].

### 2.2. Cohort Architecture

The source population comprised all unique Al-Bashir patients with any HF-related diagnosis or problem-list entry. Date of birth was audited against registration and encounter records before age was calculated. Patients younger than 18 years were excluded before the adult broad-HF cohort was formed; records with irreconcilable identifiers or dates were not analytically linked. The broad-HF cohort was contextual and was not interpreted as an HFrEF treatment denominator because it included nonspecific HF and alternative EF phenotypes.

The primary cohort comprised adults meeting a final frozen diagnosis/problem-text phenotype compatible with documented HFrEF. Structured LVEF was unavailable for systematic cohort construction; therefore, the manuscript uses “EHR-defined HFrEF phenotype,” not confirmed HFrEF. The phenotype required an HF diagnosis plus a code or normalized term indicating systolic HF, HFrEF, reduced EF, or left-ventricular systolic dysfunction. A single historical systolic-dysfunction term without later corroboration was insufficient. Generic HF entries and isolated medication use were never sufficient.

**Figure 1:**
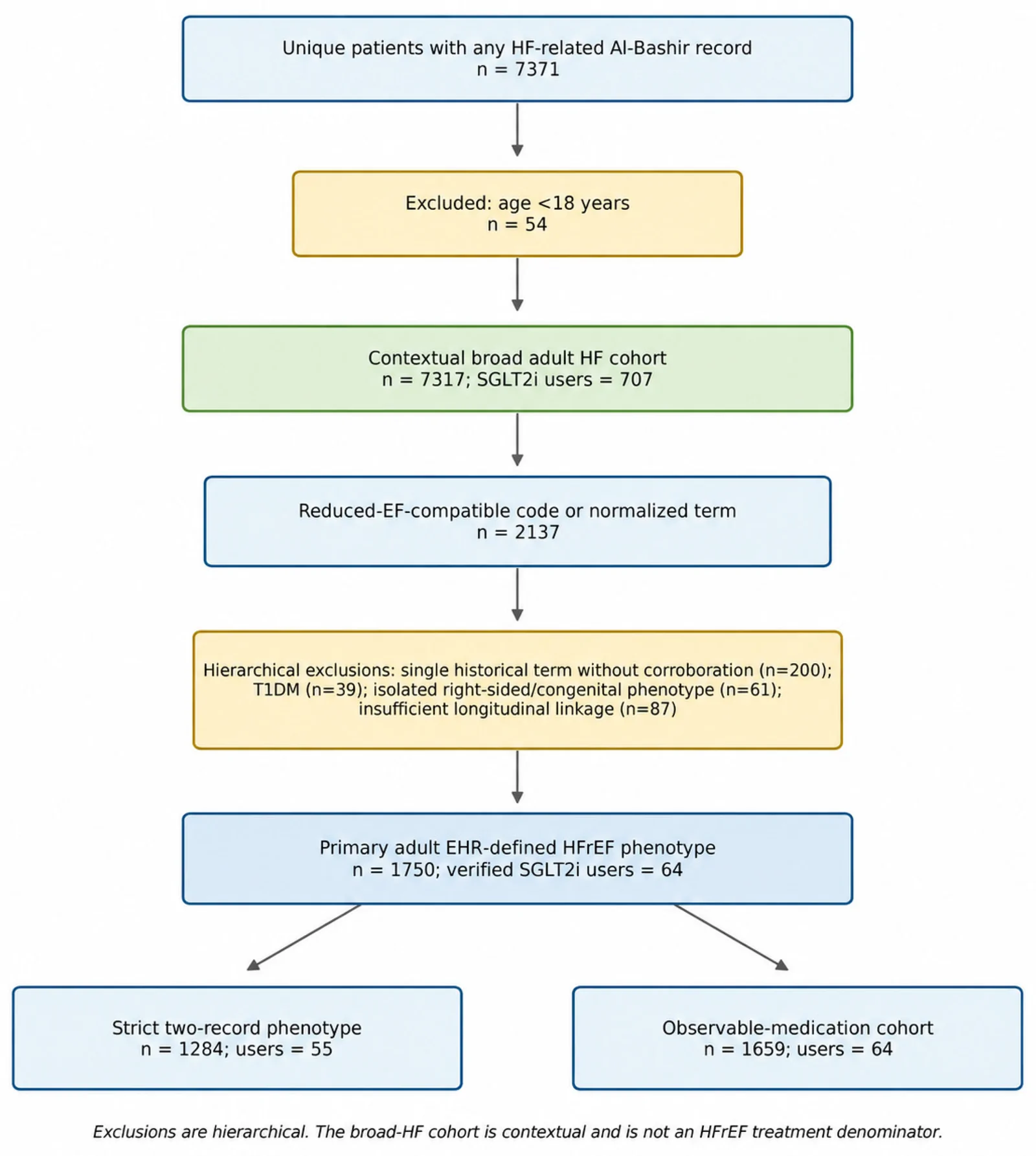
Cohort architecture and prespecified sensitivity populations.

Conflicting phenotype information was resolved hierarchically. A contemporaneous narrative LVEF > 40%, explicit HFpEF/HFmrEF diagnosis, or documented recovered/improved EF after an older reduced-EF term removed the patient from the primary cohort for the relevant index episode. The index date was the first date on which all final phenotype components were satisfied. A strict sensitivity phenotype required at least two qualifying records separated by at least 30 days, including one cardiology or HF-related discharge record. T1DM, isolated right-sided HF, congenital heart disease without qualifying left-ventricular systolic dysfunction, and records without adequate longitudinal linkage were excluded hierarchically. Figure 1 reports every cohort-derivation count. Pediatric records were audited for data quality but were not used as a treatment sensitivity analysis because pediatric evidence and labeling differ from adult HF care.

### 2.3. Phenotype Development and Internal Validation

Phenotype development and internal validation were separated. A 250-record development set, sampled before the final analysis, was used to normalize terms and define the conflict hierarchy. The dictionary and decision rules were then frozen. No term, rule, or threshold was changed after validation began.

The final phenotype was internally validated in an independent, non-overlapping simple random sample of 700 adults from the 7317-patient broad-HF cohort. None of the 700 validation records appeared in the 250-record development set. Two clinically trained reviewers, blinded to algorithm classification, independently applied a prespecified reference standard to longitudinal records. Reference-positive documented HFrEF required either a narrative or scanned LVEF ≤ 40%, or explicit cardiology/discharge documentation of HFrEF or left-ventricular systolic HF on two occasions with no later conflicting EF phenotype before index. Reference-negative status comprised LVEF > 40%, explicit HFpEF/HFmrEF/recovered EF, isolated right-sided HF, or nonspecific HF without adequate reduced-EF documentation. Disagreements were resolved by consensus with an independent senior cardiologist who did not participate in algorithm development. Because the validation sample was an unstratified simple random sample, unweighted sensitivity, specificity, positive predictive value (PPV), negative predictive value (NPV), and exact 95% CIs were appropriate. The validation remained internal to the same hospital data environment. Supplementary Tables S1 and S2 provide the diagnosis/code dictionary, normalized text terms, and conflict hierarchy, while Supplementary Table S4 provides the independent validation matrix, performance measures, and inter-rater agreement.

### 2.4. Medication Ascertainment and Validation

Medication data were obtained from active electronic orders, hospital pharmacy records, discharge medication lists, and verified medication-reconciliation entries. Generic names, brand names, strengths, and formulary identifiers were mapped using a prespecified dictionary (Supplementary Table S3). Duplicate same-day records were collapsed. Cancelled, rejected, test, or historical-only entries were excluded unless an independent active source confirmed use.

The recorded-use outcome was any verified active record of dapagliflozin or empagliflozin after cohort entry or active on the index date. Canagliflozin and ertugliflozin were captured and reported separately but did not satisfy this prespecified HFrEF treatment outcome. Sequential records for different agents were counted once at the patient level and classified separately as switching. The EHR did not reliably identify whether a prescription was issued for HF, diabetes, or CKD; accordingly, the manuscript consistently refers to recorded use among patients with the phenotype, not confirmed HF prescribing.

Medication ascertainment was compared with a reference-standard longitudinal review of all 1750 primary-cohort records. Two clinical pharmacists, each with more than five years of clinical practice and EHR medication-review experience, independently reviewed every record while blinded to the primary algorithm flag. They examined electronic orders, pharmacy issue records, discharge medication lists, medication reconciliation, discontinuation fields, and duplicate or rejected transactions. Pre-adjudication agreement and Cohen kappa were calculated. A third senior clinical pharmacist adjudicated all disagreements before database lock. This complete-cohort, double-review design avoided verification and sampling-weight bias. The observable-medication sensitivity cohort required at least one comprehensive reconciliation record plus one independent medication source.

Active 2025 beta-blocker, ARNI/ACEI/ARB, and MRA classification was additionally validated in a simple random sample of 300 of the 936 patients contributing to the standardized 2025 assessment. The same two pharmacists independently double-reviewed all 300 sampled records, and the senior pharmacist adjudicated disagreements. For each class, the validation report included the full 2 × 2 matrix, sensitivity, specificity, PPV, NPV, and exact 95% CIs (Supplementary Table S5A,B).

For annual prevalence, a medication was active when an order or pharmacy supply interval overlapped at least one day of the calendar year and had not been cancelled or rejected. When days supplied were unavailable, an outpatient order was assigned 30 days of coverage; a sensitivity analysis used 90 days. For the concurrent-GDMT analysis, the assessment date was each patient’s last HF-related encounter in 2025. A medicine was active if its explicit supply interval included that date or, when duration was missing, if it had been issued during the preceding 30 days and was not discontinued. The 90-day assumption was examined separately. In a strict documentation sensitivity analysis, a class was considered active only when an explicitly documented supply/duration interval overlapped the 2025 assessment date; no missing-duration imputation was permitted. ARNI, ACEI, and ARB exposure were also reported separately after reconciliation of overlapping renin-angiotensin-system records to a single active category at the assessment date.

### 2.5. Diabetes, Kidney Function, and Other Covariates

T2DM required either two diagnostic records on different dates or one diagnostic record plus independent supporting evidence: HbA1c ≥ 6.5%, use of a non-SGLT2 glucose-lowering medicine, or explicit specialist documentation. SGLT2i use was never used to define T2DM. T1DM was identified separately and excluded. Diabetes status was established using information available on or before the index date.

The most recent serum creatinine within 90 days before or 14 days after index was used to estimate eGFR using the 2021 race-free CKD-EPI equation [19]. Nondialysis CKD required a diagnostic code or two eGFR measurements < 60 mL/min/1.73 m^2^ separated by at least 90 days. Dialysis was analyzed separately. Measured-eGFR threshold sensitivity analyses were restricted to patients with an available eGFR. The primary threshold subset required eGFR ≥ 20 mL/min/1.73 m^2^ and no dialysis; this threshold was selected because it corresponded to the lower eGFR boundary of at least one HF-labeled study agent during 2022–2025, not because it established eligibility for a particular drug. A stricter eGFR ≥ 25 analysis assessed threshold sensitivity. Patients with missing eGFR were reported separately and were never assigned to a threshold subset. Because hypotension, acute illness, infection history, ketoacidosis risk, and patient preference were incompletely captured, neither subset was described as fully clinically eligible.

Other prespecified covariates were age, sex, hypertension, ischemic heart disease, atrial fibrillation, COPD, prior HF hospitalization, index year, and observable follow-up duration. Diagnoses were required to precede or occur on the index date. Missing laboratory values were reported explicitly and were not interpreted as normal values.

### 2.6. Outcomes

The primary temporal outcome was annual prevalence of an active SGLT2i record during each comparable calendar year, 2022–2025. For contribution to a given patient-year, a patient had to (1) satisfy the frozen HFrEF phenotype on or before the first qualifying HF-related encounter in that year; (2) have at least one HF-related encounter in the year; (3) have observable medication data during the year; and (4) not be recorded as dead, transferred, formally closed, or otherwise administratively lost before the contributed observation. Secondary outcomes were cumulative ever-recorded use during heterogeneous observable follow-up; annual prevalence by T2DM; agent distribution and switching; first recorded use after complete baseline observability; concurrent foundational GDMT at the standardized 2025 assessment date; and prespecified phenotype, medication-observability, measured-eGFR threshold, fixed-horizon, and broad-HF sensitivity analyses. Patients could contribute to more than one year.

The complete-lookback first-record cohort required at least 180 days of observable medication history before index, no active SGLT2i record during that interval or at index, and at least one post-index medication observation. Patients without the complete lookback were classified as indeterminate for first-record timing. A fixed-horizon analysis was restricted to patients with 12 months of post-index observability. Because out-of-hospital death was incompletely captured, a competing-risk model was not used; timing estimates were explicitly descriptive first-record measures.

Foundational GDMT comprised ARNI/ACEI/ARB; carvedilol, bisoprolol, or metoprolol succinate; spironolactone or eplerenone; and dapagliflozin or empagliflozin. Concurrent class counts required active overlap on the same 2025 assessment date. They did not measure adherence, persistence, dose optimization, or ingestion. Structured reasons for non-use or deferral were not systematically captured in the EHR and therefore were not retrospectively assigned or inferred from absence of a medication record.

### 2.7. Statistical Analysis

Categorical variables are presented as *n* (%), and continuous variables as mean (standard deviation) or median (interquartile range [IQR]). Exact Clopper–Pearson confidence intervals were calculated for single proportions. The T2DM contrast used a Newcombe-Wilson CI for the risk difference; the zero-event non-diabetes stratum is reported with a two-sided exact 95% CI. Signed standardized mean differences (SMDs) were calculated as SGLT2i users minus non-users using pooled standard deviations or pooled binary variances.

Annual prevalence was analyzed in 3725 patient-year observations contributed by 1722 unique patients during 2022–2025. Generalized estimating equations (GEEs) with a logit link, exchangeable working correlation, and robust standard errors accounted for repeated patients. Calendar year was modeled continuously for trend and categorically to assess nonlinearity. Because every observed outcome occurred among patients with T2DM, a prespecified T2DM-specific GEE was also fitted; a corresponding non-diabetes model was not estimable. An observable-medication annual analysis, a 90-day missing-duration assumption, and restriction to patients observed in at least two comparable years assessed robustness. A balanced-cohort sensitivity analysis additionally required the same patient to satisfy phenotype, care-engagement, medication-observability, and administrative-status criteria in all four comparable years (2022–2025); annual prevalence was then calculated using a fixed denominator, with absolute percentage-point changes reported alongside a repeated-patient GEE trend estimate. The 2020–2021 run-in counts and exact CIs were reported in Supplementary Table S6; repeated-observation trend sensitivity models and the balanced-cohort analysis are summarized in Supplementary Tables S7 and S9, respectively.

Complete separation prevented conventional estimation of the diabetes coefficient in the full cohort. The primary determinants analysis was therefore restricted to the 561 patients with T2DM, among whom 64 outcome events occurred. A Firth bias-reduced logistic model included seven prespecified parameters: age per 10 years, sex, nondialysis CKD, COPD, ischemic heart disease, index year, and observable follow-up duration. No stepwise selection was used. Confidence intervals were obtained by profile penalized likelihood, and *p* values by penalized likelihood-ratio tests. A full-cohort Firth model including T2DM was retained only as a sensitivity analysis, and its diabetes coefficient was interpreted as a separation-sensitive concentration estimate rather than a causal effect. Because index year and observable follow-up are structurally related, their Spearman correlation was quantified. Continuous-variable functional form was checked using restricted cubic-spline terms, and influence was examined using case-deletion diagnostics. To address heterogeneous exposure opportunity without including follow-up duration as a predictor, an exploratory Cox proportional-hazards sensitivity model was fitted in the complete-lookback T2DM cohort, with time from index to first recorded SGLT2i use and censoring at the last medication-observable date, recorded death/transfer/administrative closure, or 31 December 2025, whichever occurred first. Proportional-hazards assumptions were assessed using Schoenfeld residuals. All association estimates were interpreted as non-causal.

Observable follow-up was summarized for the full cohort, separately for users and non-users, and for the complete-lookback first-record cohort. The fixed 12-month proportion, based on a common follow-up horizon, was emphasized as the most directly interpretable timing estimate. As a sensitivity analysis, time to first recorded SGLT2i use in the complete-lookback cohort was estimated using Kaplan–Meier methods, with censoring at the earliest of the last medication-observable date, recorded death, transfer or administrative closure, or 31 December 2025. Because out-of-hospital death was incompletely captured, this analysis was interpreted as time to first EHR record rather than as a competing-risk estimate of clinical initiation. A conservative 12-month lower-bound sensitivity analysis treated patients without an observed first record as event-free through day 365. Missing eGFR was not imputed because renal analyses were restricted to measured values. Analyses were conducted using R version 4.5.1 with the geepack, logistf, and survival packages. Two-sided *p* values < 0.05 were considered statistically significant, with emphasis on effect estimates and confidence intervals. COPD was prespecified, but its estimate was interpreted as exploratory because only 16 treated patients had COPD, and healthcare-contact intensity was incompletely measured.

### 2.8. Ethics

The study was approved by the Institutional Review Board of Al-Bashir Hospital/Ministry of Health (IRB No. 2276; approved on 5 February 2026). Individual consent was waived for retrospective analysis of de-identified routine records. All data were processed under institutional data-governance requirements and the Declaration of Helsinki.

### 2.9. Use of Generative Artificial Intelligence in Manuscript Preparation

During the preparation of this manuscript, the authors used ChatGPT 5.5 (OpenAI) solely to assist with language editing, readability improvement, formatting refinement, and structural polishing. The tool was not used to generate original study data, conduct statistical analyses, independently interpret findings, or make decisions regarding the scientific content of the study. All AI-assisted outputs were critically reviewed, edited, and verified by the authors, who take full responsibility for the accuracy, integrity, and final content of the manuscript.

## 3. Results

### 3.1. Cohort Identification and Validation

The source query identified 7371 unique patients with any HF-related Al-Bashir record. Age verification excluded 54 patients younger than 18 years; no irreconcilable adult date-of-birth record remained. The contextual broad adult HF cohort contained 7317 patients. Hierarchical phenotype and linkage exclusions produced 1750 adults in the primary EHR-defined HFrEF cohort; 1284 met the strict two-record phenotype, and 1659 met observable-medication criteria (Figure 1).

The internally validated, independent 700-record sample contained 173 reference-positive and 527 reference-negative records and did not overlap the 250-record development set. The frozen algorithm classified 159 true positives, eight false positives, 14 false negatives, and 519 true negatives. Sensitivity was 91.91% (95% CI 86.79–95.51), specificity 98.48% (97.03–99.34), PPV 95.21% (90.78–97.91), and NPV 97.37% (95.63–98.56). Pre-adjudication inter-reviewer agreement was κ = 0.90. No algorithm rule was changed after this evaluation. Misclassifications most often involved old systolic-dysfunction terminology followed by recovered EF or narrative abbreviations absent from structured fields. The complete validation matrix and agreement summary are provided in Supplementary Table S4.

Both clinical pharmacists independently reviewed all 1750 primary-cohort medication records. They agreed before adjudication on 1744/1750 classifications (99.66%; κ = 0.95, 95% CI 0.91–0.99); the senior pharmacist adjudicated six disagreements. Against the adjudicated reference, the primary SGLT2i algorithm identified 63 true positives, one false positive, one false negative, and 1685 true negatives. The locked cohort therefore contained 64 verified users. Sensitivity was 98.44% (95% CI 91.60–99.96), specificity 99.94% (99.67–100.00), PPV 98.44% (91.60–99.96), and NPV 99.94% (99.67–100.00). The corresponding SGLT2i validation matrix is included in Supplementary Table S5A.

In the independently double-reviewed random sample of 300 active 2025 records, the beta-blocker algorithm classified 202 true positives, four false positives, eight false negatives, and 86 true negatives: sensitivity 96.2% (95% CI 92.6–98.3), specificity 95.6% (89.0–98.8), PPV 98.1% (95.1–99.5), and NPV 91.5% (83.9–96.3). Corresponding ARNI/ACEI/ARB counts were 191, five, nine, and 95: sensitivity 95.5% (91.6–97.9), specificity 95.0% (88.7–98.4), PPV 97.4% (94.1–99.2), and NPV 91.3% (84.2–96.0). MRA counts were 80, four, five, and 211: sensitivity 94.1% (86.8–98.1), specificity 98.1% (95.3–99.5), PPV 95.2% (88.3–98.7), and NPV 97.7% (94.7–99.2). Pre-adjudication agreements were 294/300 (98.0%; κ = 0.95), 292/300 (97.3%; κ = 0.94), and 296/300 (98.7%; κ = 0.96), respectively. Supplementary Tables S4, S5A and S5B provide the complete validation matrices, performance measures, and reviewer-agreement results. Full class-specific performance measures and reviewer agreement are provided in Supplementary Table S5B.

### 3.2. Patient Characteristics

The mean age was 66.0 years (SD 12.9), 988 patients (56.5%) were men, and 561 (32.1%) had T2DM. Non-dialysis CKD was present in 337 (19.3%), dialysis in 42 (2.4%), and COPD in 201 (11.5%). Median observable follow-up was 31.4 months (IQR 18.2–46.7), totaling 4582 person-years. Follow-up was 24.6 months (IQR 14.1–36.8) among users and 31.7 months (18.4–47.1) among non-users. Compared with non-users, users were older, more frequently had ischemic heart disease and COPD, entered the cohort later, and less frequently had CKD. Every user had T2DM (Table 1).

### 3.3. Primary Uptake and Diabetes-Related Difference

Sixty-four of 1750 adults had a verified SGLT2i record at any point during heterogeneous observable follow-up, an ever-recorded proportion of 3.66% (95% CI 2.83–4.65). This descriptive proportion is not a fixed-horizon risk estimate. Dapagliflozin was recorded for 42 patients (65.6%) and empagliflozin for 22 (34.4%); canagliflozin and ertugliflozin were each recorded for zero patients. No simultaneous dual-agent record or sequential switching between SGLT2i agents was identified.

All 64 users had T2DM. Uptake was 11.41% (64/561; 95% CI 8.90–14.33) among patients with T2DM and 0% among 1189 patients without diabetes (two-sided exact 95% CI 0–0.31). The Newcombe–Wilson risk difference was 11.41 percentage points (95% CI 9.01–14.31; Fisher exact *p* < 0.001). Because treatment indication was not consistently recorded, this difference represents diabetes-linked treatment concentration rather than proof that individual orders were issued for diabetes or that non-diabetic claims were rejected.

### 3.4. Comparable-Period Annual Adoption, 2022–2025

**Figure 2:**
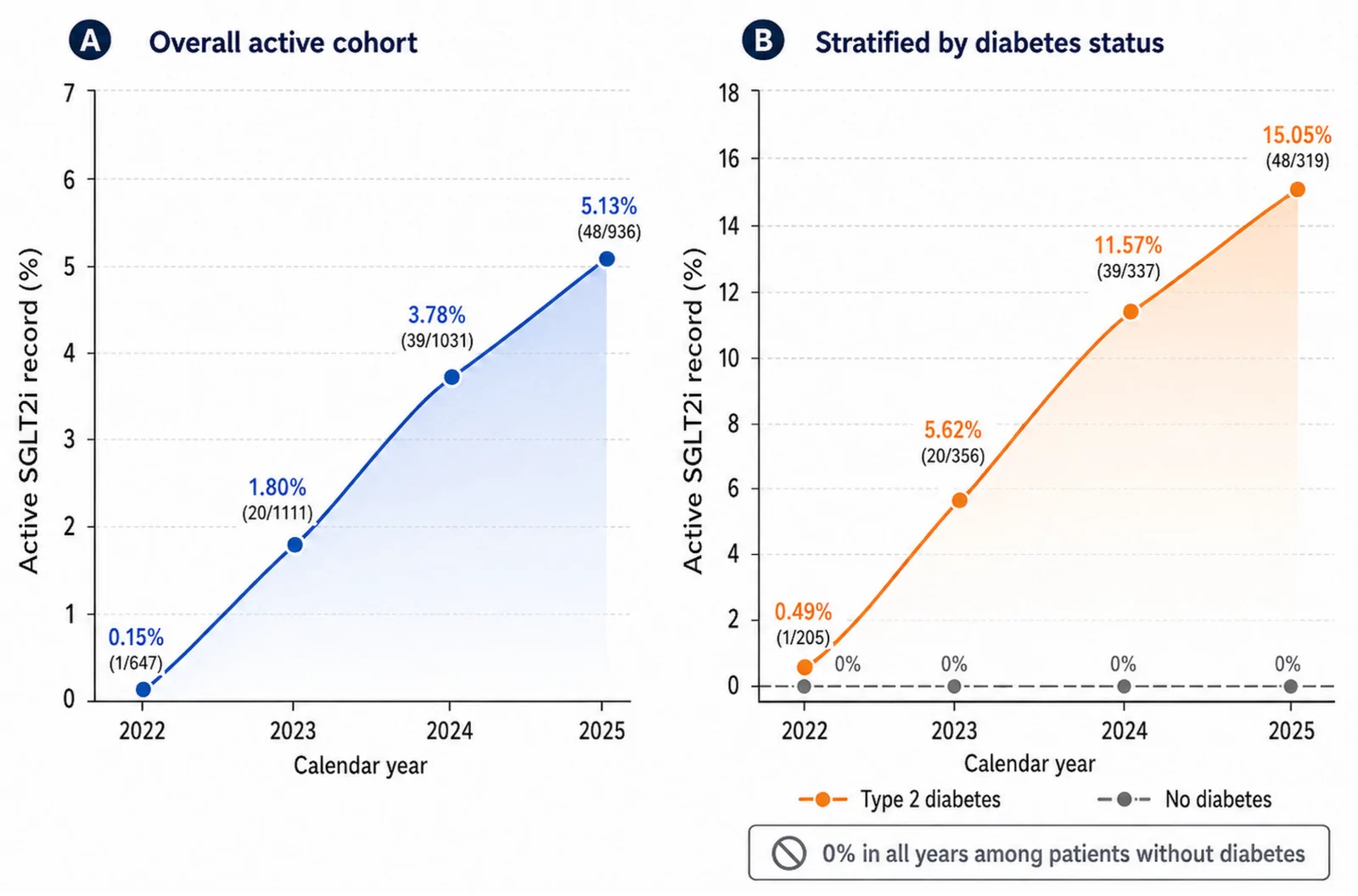
Annual prevalence of active SGLT2 inhibitor records during 2022–2025.

Across 3725 patient-year observations from 1722 unique patients, annual active-record prevalence increased from 0.15% (1/647; 95% CI 0.00–0.86) in 2022 to 5.13% (48/936; 3.81–6.74) in 2025, an absolute increase of 4.98 percentage points (Table 2; Figure 2). Each later calendar year was associated with higher odds of an active record in the patient-year GEE model (OR 1.88; 95% CI 1.59–2.23; *p* < 0.001). The categorical model indicated rapid growth through 2024 followed by a smaller absolute increase in 2025.

Among active patients with T2DM, annual prevalence rose from 0.49% in 2022 to 15.05% in 2025, an absolute increase of 14.56 percentage points. The T2DM-specific repeated-patient GEE gave an OR of 1.93 per later calendar year (95% CI 1.62–2.31; *p* < 0.001). No active record was observed among patients without diabetes in any comparable year, so a stratum-specific trend model was not estimable. Medication reconciliation or a linked independent medication source was available for 91.8%, 94.1%, 95.0%, and 95.7% of active patients in 2022–2025, respectively. Restriction to those observable-medication records produced annual estimates of 0.17%, 1.91%, 3.98%, and 5.36%, with a similar trend (OR 1.86; 95% CI 1.56–2.21). Among 1091 patients observed in at least two comparable years (3094 patient-years), the annual trend remained consistent (OR 1.82; 95% CI 1.51–2.20; *p* < 0.001). In the balanced four-year sensitivity cohort (*n* = 468), prevalence increased from 0.21% (1/468) in 2022 to 1.71% (8/468) in 2023, 3.85% (18/468) in 2024, and 5.34% (25/468) in 2025, corresponding to an absolute increase of 5.13 percentage points and a repeated-patient OR of 2.04 per calendar year (95% CI 1.50–2.78; *p* < 0.001). Within the stable T2DM subgroup (*n* = 158), prevalence increased from 0.63% to 15.82% (absolute increase 15.19 percentage points), whereas all 310 stable patients without diabetes remained at 0%. These sensitivity results suggest that the upward temporal pattern would not be explained solely by changing annual cohort composition. The 2020–2021 run-in denominators and completeness metrics appear in Supplementary Table S6, repeated-patient trend sensitivities in Table S7, and the stable-cohort analysis in Table S9.

### 3.5. First Recorded Use in the Complete-Lookback Cohort

Among all 64 verified users, seven had prevalent use at index, 50 met the complete-lookback definition for first recorded post-index use, and seven had insufficient baseline history and were classified as indeterminate. The complete-lookback first-record cohort contained 1402 patients with at least 180 days of baseline medication observability, no baseline SGLT2i use, and post-index medication follow-up. Median follow-up in this cohort was 29.8 months (IQR 17.0–44.6).

In the verified fixed-horizon cohort, 27 of 1268 patients with 12 months of post-index observability had a first recorded SGLT2i use within one year (2.13%; 95% CI 1.41–3.08); this estimate was emphasized because every patient had the same observation horizon. Among the 50 patients who eventually had a first post-index record, the conditional median time from index was 322 days (IQR 91–602), with 8, 13, 19, and 27 first records observed within 30, 90, 180, and 365 days, respectively. The 322-day value therefore describes timing only among eventual users and is not a median waiting time for the full cohort. In the Kaplan–Meier sensitivity analysis of all 1402 complete-lookback patients, estimated first-record probabilities were 0.57% at 30 days, 0.94% at 90 days, 1.38% at 180 days, 2.08% at 365 days, and 3.72% at 730 days. At 12 months, the censoring-based estimate (2.08%) was close to both the verified fixed-horizon estimate (2.13%) and the conservative lower-bound estimate that treated all 1402 patients as event-free through day 365 unless a first record was observed (27/1402; 1.93%). These analyses concern first EHR-recorded use and do not establish dispensing, ingestion, persistence, or initiation outside the system; censoring assumptions and landmark estimates are summarized in Supplementary Table S10.

### 3.6. Concurrent Foundational GDMT at the 2025 Assessment

**Figure 3:**
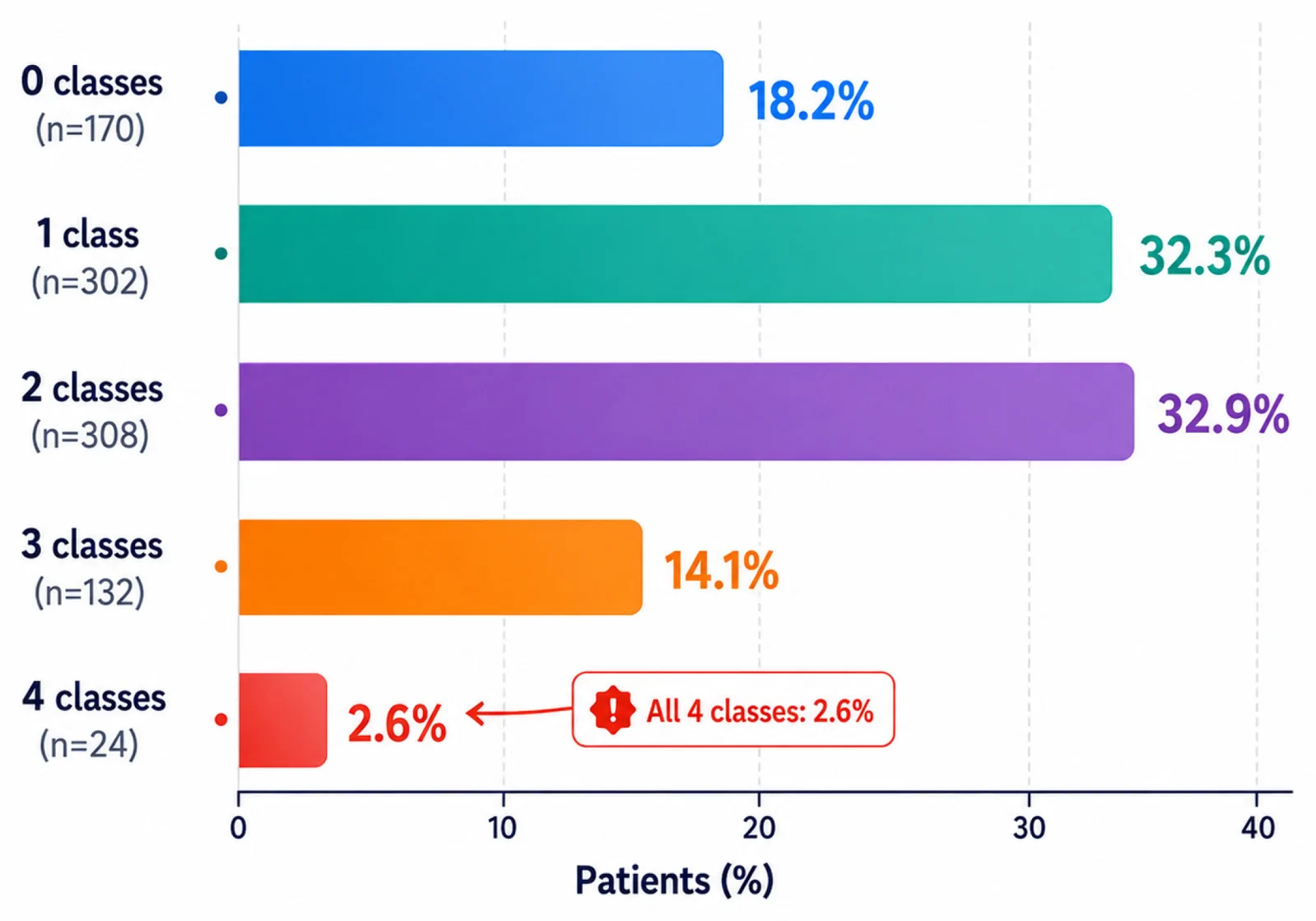
Number of concurrently active foundational classes for the 2025 assessment.

Among 936 patients active in care during 2025, the assessment-date algorithm identified concurrent active records for an evidence-based beta-blocker in 652 (69.7%), ARNI/ACEI/ARB in 500 (53.4%), MRA in 210 (22.4%), and SGLT2i in 48 (5.1%). The 500 renin-angiotensin-system records comprised ARNI in 164 patients (17.5%), ACEI in 221 (23.6%), and ARB in 115 (12.3%) after reconciliation of overlapping records. Twenty-four patients (2.6%) had all four classes concurrently active, while 170 (18.2%) had none (Table 3; Figure 3). Among the 48 SGLT2i users, 24 had four concurrent classes, 12 had three, eight had two, and four had SGLT2i alone. In the documentation audit, 1131 of 1410 positive patient-class classifications (80.2%) were supported by an explicit duration or supply interval, whereas 279 (19.8%) relied on the 30-day missing-duration rule. Requiring directly documented coverage without imputation reduced the class prevalences to 55.3% for beta-blockers, 41.6% for ARNI/ACEI/ARB, 15.9% for MRA, and 3.8% for SGLT2i, with 14/936 patients (1.5%) having all four classes concurrently documented. Using the 90-day missing-duration assumption yielded 27/936 (2.9%), with four concurrent classes. Reasons for non-use were not systematically captured and therefore were not retrospectively assigned. Full documentation sensitivity results are provided in Supplementary Table S12. These values do not establish dispensing, adherence, dose optimization, or ingestion.

### 3.7. Determinants Among Patients with T2DM

**Figure 4:**
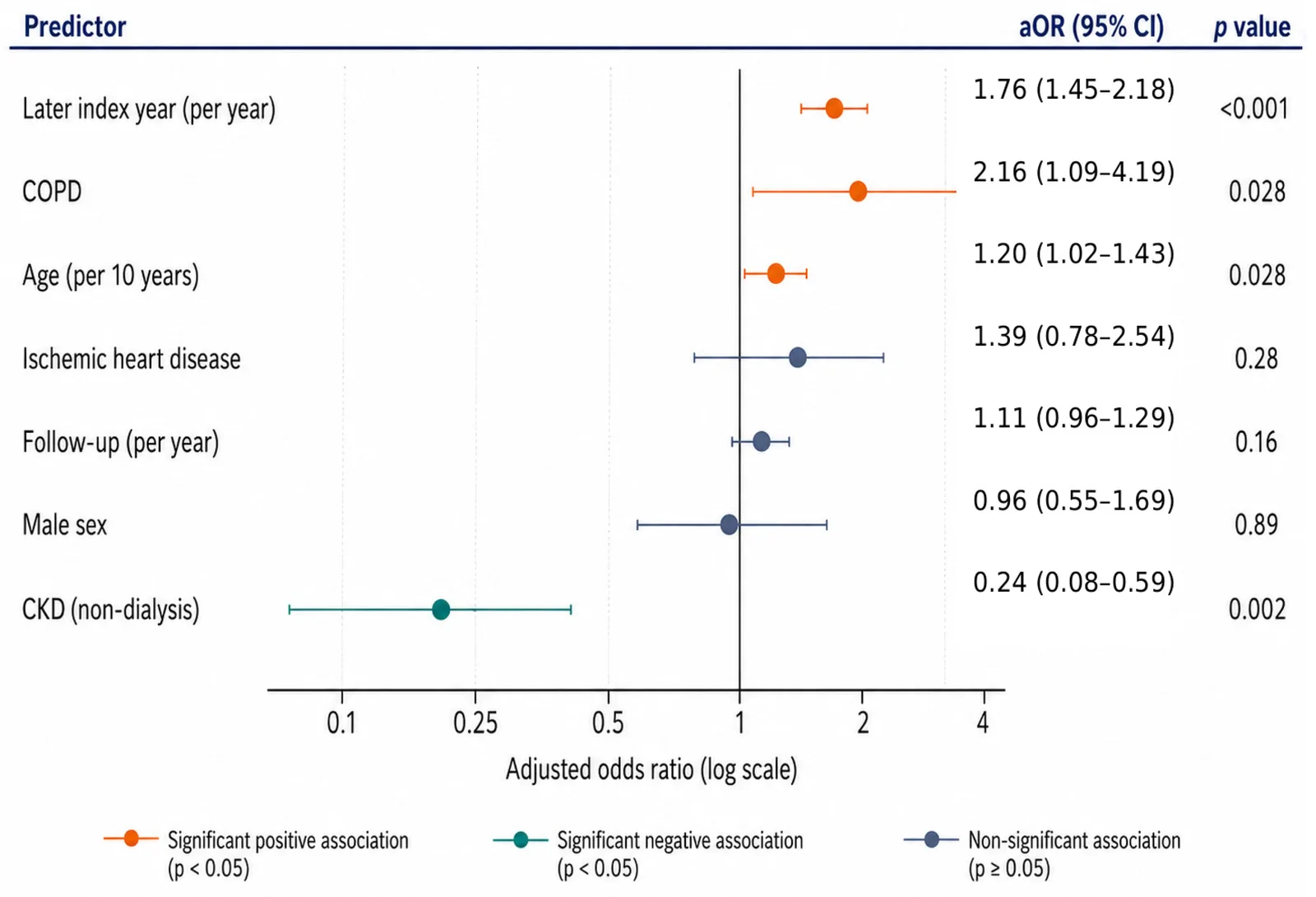
Adjusted associations with recorded SGLT2 inhibitor use among patients with T2DM.

The primary determinants model was restricted to 561 patients with T2DM, including 64 users. Later index year and older age were associated with higher odds of recorded use in the Firth model, while an inverse estimate was observed for nondialysis CKD (Table 4; Figure 4). Because only four treated patients had nondialysis CKD, that estimate was considered sparse-data, exploratory, and non-causal rather than evidence of an independent renal treatment effect. The COPD estimate was likewise treated as exploratory because it was based on 16 treated patients with COPD and could reflect healthcare-contact intensity. Additional diagnostics showed that index year and observable follow-up were moderately inversely correlated (Spearman ρ = −0.61; *p* < 0.001), the maximum variance inflation factor was 2.18, restricted cubic-spline terms did not improve fit for age (*p* = 0.47) or follow-up duration (*p* = 0.33), and no single case changed the direction of any coefficient in case-deletion influence analyses. In the complete-lookback T2DM Cox sensitivity model (*n* = 462; 50 first-record events), later index year remained associated with earlier first recorded use (hazard ratio 1.52 per year; 95% CI 1.18–1.95; *p* = 0.001), whereas the CKD estimate remained inverse but imprecise (hazard ratio 0.32; 95% CI 0.11–0.80; *p* = 0.016). The global proportional-hazards test was not significant (*p* = 0.68). These sensitivity analyses are summarized in Supplementary Table S11 and do not establish causality.

In the full-cohort Firth sensitivity model, T2DM produced an aOR of 289.4 (95% CI 40.2–2082.6), reflecting complete separation. This coefficient was not interpreted as a causal effect. Later year and inverse associations with kidney disease remained directionally consistent (Supplementary Table S8).

### 3.8. Kidney Function and Measured-eGFR Threshold Sensitivity Analyses

eGFR was available for 1456 patients. Recorded use occurred in 50/860 patients with eGFR ≥ 60 mL/min/1.73 m^2^, 8/476 with eGFR 30–59, 0/42 with eGFR 25–29, 1/28 with eGFR 20–24, 0/8 with eGFR < 20, and 0/42 receiving dialysis. Five of 294 patients with missing eGFR had a verified record; those 294 were excluded from measured-eGFR threshold analyses rather than assumed eligible.

The measured-eGFR subset compatible with at least one HF-labeled agent (eGFR ≥ 20 and no dialysis) contained 1406 patients and 59 users (4.20%; 95% CI 3.21–5.38). The stricter eGFR ≥ 25 subset contained 1378 patients and 58 users (4.21%; 3.21–5.41). No user without diabetes was identified in either subset. These analyses address renal measurement and label thresholds only; they do not establish complete clinical eligibility or reasons for non-use.

### 3.9. Sensitivity Analyses

The principal diabetes-related pattern was unchanged across clinically relevant sensitivity cohorts (Table 5). The all-age analysis was removed because pediatric treatment evidence and labeling differ from adult HF care. No user without diabetes was identified in the strict phenotype, observable-medication, complete-lookback first-record, fixed-horizon, or measured-eGFR threshold analyses.

The broad adult HF cohort produced a higher contextual estimate of 9.66% (707/7317). Of its 707 users, 64 met the adult HFrEF-compatible phenotype; 130 had documentation inconsistent with that phenotype, including preserved/mildly reduced EF terminology where available; and 513 had only nonspecific HF documentation. The broad estimate therefore represents SGLT2i use among adults with any HF-related record and should not be interpreted as HFrEF-specific uptake.

## 4. Discussion

### 4.1. Principal Findings and Adoption Trajectory

This study identifies a substantial implementation gap between contemporary HFrEF treatment recommendations and recorded medication use in a major Jordanian public-hospital EHR. Only 64 of 1750 adults meeting the internally validated EHR-defined HFrEF phenotype had a verified SGLT2i record at any point during observable follow-up, corresponding to a cumulative ever-recorded proportion of 3.66%. Because follow-up duration was heterogeneous, this value should not be interpreted as a fixed-horizon treatment probability. The annual analysis is more informative for temporal adoption: active-record prevalence increased from 0.15% in 2022 to 5.13% in 2025, an absolute increase of 4.98 percentage points, and the repeated-patient model showed a strong calendar-year association. Importantly, the balanced four-year cohort produced a closely similar absolute increase (0.21% to 5.34%; +5.13 percentage points), suggesting that the temporal pattern would persist even with a fixed annual denominator. Thus, the principal message is not that adoption was static, but that meaningful diffusion occurred from an extremely low baseline and still left most patients without recorded treatment by 2025.

The trajectory also suggests that guideline publication and accumulating trial evidence were followed by gradual rather than immediate implementation. SGLT2i are distinctive among the four foundational HFrEF classes because the heart-failure dose is fixed and does not require serial up-titration. Consequently, a persistent implementation gap cannot be explained solely by the dose-optimization barriers that complicate beta-blocker, renin-angiotensin system, and MRA therapy. The remaining gap is more compatible with a combination of treatment recognition, clinical eligibility assessment, formulary access, reimbursement, prescriber workflow, and follow-up processes. The study cannot determine the relative contribution of these mechanisms, but the pattern provides a clear target for implementation research.

Several design features increase confidence that the low observed uptake is not simply an artifact of one weak data field. Phenotype development was separated from validation of a frozen algorithm in an independent, non-overlapping sample; SGLT2i ascertainment was checked against complete-cohort longitudinal medication review; annual inference was restricted to years in which medication-source completeness exceeded 90%; and the main pattern remained unchanged across strict-phenotype, observable-medication, fixed-horizon, and measured-eGFR sensitivity cohorts. These safeguards do not eliminate missing external prescriptions or uncertainty regarding treatment indication, but they make it less likely that the central diabetes-stratified pattern is explained by routine EHR misclassification alone.

### 4.2. Relationship to Previous Jordanian and Regional Evidence

The present findings should be interpreted alongside, rather than directly pooled with, the Jordan Heart Failure Registry. The registry reported SGLT2i use in 9.0% of 2151 patients recruited across 21 centers during 2021–2023 [16]. Its denominator included acute and chronic HF across ejection-fraction phenotypes, whereas the present primary cohort required documentation compatible with HFrEF and was derived from one public tertiary hospital. Notably, the broad adult HF cohort in the present dataset yielded a contextual estimate of 9.66%, close to the registry estimate, while the more specific HFrEF-compatible cohort yielded 3.66%. This convergence at the broad-HF level, together with divergence after phenotype restriction, illustrates how case definition, site mix, medication ascertainment, and observation window can materially change apparent uptake. Neither estimate should therefore be treated as a national HFrEF prevalence without attention to the underlying denominator.

A separate analysis of the Jordanian registry reported complete four-pillar therapy in only 0.6% of participants and similarly identified SGLT2i as the least frequently used component [20]. The 2.6% concurrent four-class estimate in the present 2025 assessment is numerically higher, which is directionally compatible with improving adoption over time, but the two values are not directly comparable. The earlier study included a broader HF population, used a different operational definition of the four-pillar regimen, and assessed medication exposure in a multicenter registry rather than active overlap on a standardized EHR assessment date. The more defensible interpretation is that both Jordanian datasets indicate a major residual gap in comprehensive disease-modifying therapy, while the current longitudinal data suggest that the gap may be narrowing.

The patient-level pattern also complements the recent Jordanian mixed-methods study of treating and insurance physicians [17]. In that study, 61.2% of insurance physicians classified SGLT2i as diabetes-only medicine, 67.8% of treating physicians described prior authorization as a major barrier, and 59.3% reported authorization-related delays exceeding four weeks. The complete absence of recorded use among patients without diabetes in the present cohort and the long interval to first recorded use are compatible with the previously reported implementation barriers. Importantly, the two studies answer different questions: the stakeholder study identifies perceived and reported mechanisms, whereas the present analysis demonstrates how treatment concentration appears in longitudinal patient records. Their consistency strengthens the case for prospective investigation of reimbursement and indication coding but does not prove that any individual patient in this cohort was denied treatment because of a specific policy or misconception.

Regional comparison further emphasizes the importance of health-system context. A 2026 Saudi multicenter cohort of patients receiving longitudinal tertiary HF care reported SGLT2i use in 57.9%, with diabetes independently associated with treatment and advanced CKD associated with lower prescribing [15]. The magnitude is far higher than in the present public-hospital cohort, but the Saudi population was drawn from specialized tertiary cardiac centers, extended through 2024, and had different medication-access and care pathways. The contrast should therefore be viewed as evidence of achievable implementation under a different service model rather than as a direct performance ranking between countries.

Ischemic heart disease was present in 43.2% of the present cohort, making the evolving post-myocardial-infarction evidence contextually relevant. A recent review by Buonpane and colleagues [21] concluded that randomized post-acute-myocardial-infarction studies have not shown consistent reductions in recurrent ischemic coronary events despite favorable signals for heart-failure-related outcomes, ventricular remodeling, and cardiometabolic parameters. This distinction is important here: the high ischemic burden may shape clinical complexity and implementation priorities, but it should not be used to imply a post-myocardial-infarction indication beyond current guideline-supported heart-failure indications.

### 4.3. Diabetes-Linked Treatment Concentration

The most clinically distinctive finding was the complete concentration of verified SGLT2i records among patients with T2DM. Uptake was 11.41% among 561 patients with T2DM and 0% among 1189 patients without diabetes; the exact upper 95% confidence limit in the non-diabetes stratum was 0.31%. Randomized HFrEF evidence has demonstrated benefit with dapagliflozin and empagliflozin irrespective of diabetes status [6,7,8,9,10]. Therefore, diabetes should no longer function as the principal conceptual boundary for SGLT2i use in HFrEF. The persistence of a zero non-diabetes cell through 2025, and across multiple sensitivity cohorts, indicates that the diabetes-independent HF indication had not translated into the recorded treatment pattern at this site.

This observation must nevertheless be framed as treatment concentration rather than proof of prescriber intent. The EHR did not reliably encode whether a given order was issued for HF, diabetes, or CKD, and external prescriptions may have been missed. Some or all of the 64 treated patients may have received SGLT2i principally for glycemic or renal indications. Conversely, some patients without diabetes may have obtained treatment outside the hospital system. Even with those caveats, the complete separation is stronger than the more modest diabetes gradients reported internationally. In the US GWTG-HF cohort, SGLT2i discharge prescription was 26.2% in patients with T2DM versus 15.5% in those without T2DM [14], while the Saudi study also found diabetes independently associated with use [15]. These data suggest that the historical diabetes identity of the drug class continues to influence prescribing across settings, but the Jordanian pattern observed here is unusually pronounced and warrants direct policy and workflow investigation.

### 4.4. Timing of First Recorded Use

The first-record analysis adds a temporal dimension that is not captured by prevalence alone, but the population-level and conditional measures must be separated. The most directly interpretable verified result is that only 27 of 1268 patients with a common 12-month observation horizon (2.13%) had a first recorded SGLT2i use within one year. The Kaplan–Meier sensitivity estimate at one year was similar (2.08%), as was the conservative lower bound using all 1402 complete-lookback patients as the denominator (1.93%). By contrast, the 322-day median was calculated only among the 50 patients who eventually had a first post-index record. It therefore describes the distribution of event times among eventual users and cannot estimate the typical delay for the full eligible cohort.

These findings support describing low observed first-record uptake within the first year rather than assigning a cohort-wide measure of therapeutic inertia. EVOLUTION HF documented delayed initiation of newer therapies in several health systems [22], but its definitions and observation structure differ from the present EHR analysis, so the 322-day conditional median should not be directly compared as though it represented the waiting time of all patients. In the present data, the robust inference is the small proportion with a documented first record during a common 12-month horizon, with similar results under alternative censoring assumptions. Incomplete capture of out-of-hospital death remains important because the Kaplan–Meier sensitivity analysis estimates time to the first record observed in Hakeem, not a competing-risk probability of clinical initiation.

The practical implication is that adoption should be assessed not only as a cross-sectional proportion but also using prespecified time-to-action metrics with transparent observability requirements. Future quality programs could report the proportion of patients with documented HFrEF who have an SGLT2i record before discharge or within 30 and 365 days of a qualifying encounter, while separately recording clinical contraindication, temporary deferral, patient preference, and access barriers. Such metrics would be more interpretable than a conditional median calculated only among eventual users.

### 4.5. Chronic Kidney Disease: An Exploratory Signal

An inverse Firth-model estimate was observed for nondialysis CKD among patients with T2DM (adjusted OR 0.24, 95% CI 0.08–0.59), but only four treated patients had nondialysis CKD. This sparse event count substantially limits precision and makes the estimate sensitive to residual differences in treatment opportunity and clinical eligibility. It should therefore be regarded as an exploratory signal for audit rather than evidence of an independent CKD-related prescribing effect. The EHR did not systematically capture contemporaneous blood pressure, acute kidney injury, volume status, intercurrent illness, ketoacidosis risk, infection history, or clinician-documented contraindications, so appropriate clinical deferral cannot be separated from potentially avoidable underuse.

The direction of this exploratory signal has parallels in other settings. In GWTG-HF, SGLT2i discharge prescription was lower among patients with CKD than among those without CKD (18.6% versus 21.8%) despite eligibility criteria that excluded eGFR < 20 mL/min/1.73 m^2^ [14]. In the Saudi cohort, advanced CKD with eGFR < 30 mL/min/1.73 m^2^ was associated with markedly lower prescribing (adjusted OR 0.26) [15]. These comparisons support the relevance of renal complexity to implementation, but they do not validate the magnitude of the present estimate, which is based on only four treated CKD patients and remains hypothesis-generating.

The measured-eGFR sensitivity analyses are informative but should not be over-read. Overall recorded use remained only about 4.2% among patients with eGFR ≥ 20 or ≥25 mL/min/1.73 m^2^ and no dialysis, and no patient without diabetes was treated in either threshold cohort. These analyses show that low uptake was not eliminated simply by excluding patients below common renal initiation thresholds; they do not establish complete clinical eligibility or prove that the CKD association itself persisted after restriction. A prospective audit should therefore capture contemporaneous eGFR and acute kidney injury, blood pressure, volume status, acute illness, prior ketoacidosis, recurrent genitourinary infection, patient preference, prescriber-documented contraindications, and the specific renal threshold used in treatment decisions.

### 4.6. Four-Pillar GDMT and Multilevel Clinical Implications

The broader GDMT analysis indicates that SGLT2i underuse occurred within a wider implementation problem. Suboptimal implementation of foundational HFrEF therapy has also been documented in large outpatient registries [23]. At the standardized 2025 assessment, 69.7% had an evidence-based beta-blocker record, 53.4% had ARNI/ACEI/ARB, 22.4% had an MRA, and only 5.1% had an SGLT2i. Consequently, just 2.6% had all four foundational classes concurrently active, increasing to 2.9% under the 90-day missing-duration assumption. In the strict documentation sensitivity analysis, 80.2% of positive patient-class classifications had an explicit duration/supply interval and the four-pillar estimate fell to 1.5% when only directly documented coverage was accepted. The renin-angiotensin-system split was 17.5% ARNI, 23.6% ACEI, and 12.3% ARB. These documentation sensitivities reinforce that the exact four-pillar percentage depends on medication-record assumptions, while the qualitative conclusion of substantial under-implementation remains unchanged. The primary 2.6% estimate is below the 9.4% quadruple-therapy discharge rate reported in the 2021–2022 GWTG-HF cohort [14] and is numerically higher than the 0.6% four-pillar use previously reported from the Jordan registry [20]. Because the studies differ in population and exposure definition, these comparisons should be interpreted as contextual rather than as precise performance rankings.

The very low four-class overlap matters because the four therapies are complementary rather than interchangeable. Modeling studies suggest substantial survival gains when comprehensive disease-modifying therapy is implemented [24,25], while the contemporary clinical strategy has shifted from slow sequential escalation toward early initiation of all foundational classes when clinically feasible [4,26,27]. The present data do not establish whether missing classes reflected contraindication, intolerance, prior treatment failure, or access barriers, and they do not measure target-dose achievement. They nevertheless indicate that a large proportion of patients were not recorded as receiving a complete foundational regimen at the same point in care.

At the clinician level, every confirmed or strongly documented HFrEF encounter should trigger a structured review of the four foundational classes, with explicit documentation of current therapy, renal function, blood pressure, contraindications, prior intolerance, and the reason for any omitted class. Because SGLT2is require no dose titration for HF, the decision should be framed as initiation versus documented deferral rather than placement at the end of a traditional titration sequence. Reassessment after transient acute illness or volume instability is essential so that a temporary reason for non-initiation does not become permanent therapeutic inertia.

Clinical pharmacists can operationalize this process. A pharmacist-led HF medication service could perform admission and discharge reconciliation, identify missing foundational classes, verify renal and volume-status parameters, provide sick-day and adverse-effect counseling, prepare prior-authorization documentation, and arrange early post-discharge medication review. Pharmacists can also distinguish a true contraindication from a legacy warning, reconcile prescriptions obtained outside the hospital, and track refill or dispensing gaps. Although STRONG-HF did not test an SGLT2i-specific pharmacist intervention, it supports the broader principle that protocolized, high-intensity post-discharge review can accelerate GDMT optimization and improve outcomes [28].

At the hospital level, the findings support HFrEF-specific order sets and discharge bundles that display SGLT2i as HF therapy independent of diabetes, mandatory indication fields, a standardized reason-for-non-use menu, and automated reassessment prompts. A dashboard should report not only overall SGLT2i prevalence but also diabetes-stratified use, initiation within 30 days, four-pillar completion, CKD-stratified use, and documented reasons for deferral. Such measures would help distinguish clinical contraindication from workflow failure and would allow service lines to detect whether improvement is occurring equitably across patient groups.

At the payer and formulary level, authorization criteria should explicitly recognize HFrEF as an indication independent of diabetes and should avoid requiring diabetes coding as a proxy for eligibility. Standardized electronic authorization, acceptance of cardiology or validated HFrEF documentation, transparent renal thresholds, and rapid appeal pathways could reduce avoidable delay. At the national level, the Ministry of Health, major payers, hospital formularies, and professional societies could align coverage criteria with current HF recommendations, define a minimum dataset for HFrEF quality audit, and use multicenter benchmarking to identify unwarranted variation. The earlier Jordanian stakeholder findings make reimbursement and indication classification particularly plausible targets for system redesign [17]. The principal implementation barriers identified or plausibly suggested by the current findings, together with corresponding actionable responses, are summarized in Table 6.

### 4.7. Research Agenda

The next research step should move from documenting underuse to identifying where the treatment pathway fails. First, multicenter studies should link Hakeem prescribing records with hospital pharmacy dispensing, external community-pharmacy or claims data where available, and payer authorization outcomes. This would separate clinician intent from successful dispensing and would quantify how much apparent non-use reflects care outside the hospital. Second, longitudinal analyses should distinguish initiation, dispensing, refill persistence, discontinuation, switching, and re-initiation rather than treating any historical medication record as equivalent exposure. Third, reasons for non-initiation should be prospectively coded, including renal or hemodynamic instability, infection concerns, patient preference, affordability, prescriber deferral, formulary restriction, and authorization denial.

Fourth, implementation studies should evaluate pharmacist–cardiologist pathways, EHR decision support, discharge bundles, and payer-policy changes using prespecified outcomes such as initiation within 30 days, four-pillar completion, time to authorization, documented deferral reasons, and 6- and 12-month persistence. Fifth, once prescribing and dispensing exposure can be measured reliably, outcome studies should evaluate HF hospitalization, kidney outcomes, and mortality using time-updated treatment definitions and appropriate control of confounding. A national or multicenter Jordanian HFrEF implementation registry would be particularly valuable because it could establish whether the extreme diabetes-linked concentration observed at this hospital is site-specific or reflects a broader health-system pattern.

### 4.8. Strengths and Limitations

The study has several strengths relevant to interpretation. It uses a large patient-level source population, a frozen HFrEF-compatible phenotype evaluated in an independent non-overlapping validation sample, complete-cohort double-reviewed SGLT2i ascertainment, independently defined diabetes status, explicit auditing of medication-source completeness, repeated-patient annual analysis, a clean-lookback first-record cohort, a fixed 12-month horizon, concurrent GDMT assessment, rare-event regression, and prespecified phenotype, observability, and renal-threshold sensitivity analyses. The design therefore addresses a different and complementary question to the prior Jordanian registry and stakeholder studies: how recorded SGLT2i adoption evolved longitudinally within a defined public-hospital EHR population.

Important limitations constrain causal interpretation. First, structured LVEF was unavailable for systematic cohort construction, so the primary phenotype represents documented HFrEF rather than echocardiographically confirmed HFrEF in every patient. Manual validation reduced misclassification, but the reference standard partly relied on overlapping diagnostic and narrative sources, creating potential incorporation bias, and validation was internal to one hospital. Second, an active EHR medication record does not establish treatment indication, community dispensing, medication possession, ingestion, adherence, or persistence; prescriptions originating outside the participating system may be missed. Third, the study cannot establish that all untreated patients were clinically eligible. Blood pressure, acute kidney injury, acute illness, volume status, infection history, ketoacidosis risk, affordability, patient preference, prescriber specialty, structured reasons for non-use, and detailed reimbursement decisions were incompletely captured. The strict documented-coverage sensitivity analysis addresses one source of medication-record uncertainty but does not resolve clinical eligibility.

Fourth, only 64 patients had verified recorded use, which limited model complexity, precision, and interaction testing. The CKD estimate was based on only four treated patients with nondialysis CKD and should be regarded as an exploratory signal rather than an independent causal association; the positive COPD estimate is also exploratory. Index year and observable follow-up were structurally related, although the diagnostics and time-to-event sensitivity analysis suggested that the later-calendar-year pattern was not solely explained by exposure opportunity. Fifth, cumulative ever-recorded use reflects heterogeneous follow-up; the annual 2022–2025 analyses, verified fixed-horizon cohort, and balanced four-year cohort provide more comparable temporal estimates. Sixth, out-of-hospital death was incompletely captured, so first-record timing cannot be interpreted as a competing-risk estimate of treatment initiation. The verified fixed-horizon proportion and alternative censoring analyses were therefore emphasized over the 322-day median among eventual users. Seventh, the broad-HF analysis is contextual and should not be interpreted as HFrEF-specific uptake. Finally, clinical outcomes were deliberately not analyzed because the study evaluates recorded medication implementation rather than treatment effectiveness. These limitations mean that the results define the size and pattern of an implementation gap but do not identify a single causal explanation for it.

## 5. Conclusions

Recorded SGLT2i use increased from 2022 to 2025 but remained low among adults with an EHR-defined HFrEF phenotype internally validated in an independent, non-overlapping sample at a major Jordanian public hospital. The verified annual absolute increase was 4.98 percentage points, and the stable-cohort sensitivity analysis showed a closely similar trajectory, suggesting that changing annual cohort composition would not fully explain the temporal pattern. All identified users had T2DM, while no use was recorded among 1189 patients without diabetes. The verified 12-month fixed-horizon estimate showed first recorded use in only 2.13% of patients with complete one-year observability. These descriptive disparities are compatible with incomplete translation of contemporary HFrEF therapy into routine practice but do not identify a causal mechanism. Multicenter linkage of prescribing, dispensing, clinical eligibility, reimbursement, and structured reasons for non-use is required, alongside prospective evaluation of multidisciplinary initiation pathways.

## Figures and Tables

**Table 1:** Baseline characteristics by recorded SGLT2 inhibitor use.

Characteristic	Overall (*n* = 1750)	SGLT2i Use (*n* = 64)	No SGLT2i Use (*n* = 1686)	SMD
Age, years, mean (SD)	66.0 (12.9)	70.2 (10.9)	65.8 (13.0)	0.35
Age ≥ 65 years	946 (54.1)	43 (67.2)	903 (53.6)	0.28
Male sex	988 (56.5)	36 (56.3)	952 (56.5)	−0.01
Type 2 diabetes	561 (32.1)	64 (100.0)	497 (29.5)	2.19
Hypertension	1278 (73.0)	50 (78.1)	1228 (72.8)	0.12
Ischemic heart disease	756 (43.2)	42 (65.6)	714 (42.3)	0.48
Atrial fibrillation	421 (24.1)	13 (20.3)	408 (24.2)	−0.09
Non-dialysis CKD	337 (19.3)	4 (6.3)	333 (19.8)	−0.41
Dialysis	42 (2.4)	0 (0.0)	42 (2.5)	−0.23
COPD	201 (11.5)	16 (25.0)	185 (11.0)	0.37
Prior HF hospitalization	694 (39.7)	32 (50.0)	662 (39.3)	0.22
eGFR, mL/min/1.73 m^2^, mean (SD); measured n	67.8 (25.2); 1456	78.4 (18.8); 59	67.1 (25.5); 1397	0.50
Missing eGFR	294 (16.8)	5 (7.8)	289 (17.1)	−0.29
Index year 2023–2025	1200 (68.6)	58 (90.6)	1142 (67.7)	0.59
Observable follow-up, months, median (IQR)	31.4 (18.2–46.7)	24.6 (14.1–36.8)	31.7 (18.4–47.1)	—

Note: Values are *n* (%) unless otherwise indicated. Measured eGFR denominators are shown for each column. Signed SMDs are calculated as users minus non-users; an SMD was not calculated for medians. CKD, chronic kidney disease; COPD, chronic obstructive pulmonary disease; eGFR, estimated glomerular filtration rate; HF, heart failure; IQR, interquartile range; SD, standard deviation; SGLT2i, sodium-glucose cotransporter-2 inhibitor; SMD, standardized mean difference.

**Table 2:** Annual active-cohort SGLT2 inhibitor prevalence during the comparable-data period.

Year	Active Cohort, *N*	Overall Users, *n* (%; 95% CI)	T2DM, *N*	T2DM Users, *n* (%; 95% CI)	No T2DM, *N*	No T2DMUsers, *n* (%; 95% CI)
2022	647	1 (0.15; 0.00–0.86)	205	1 (0.49; 0.01–2.69)	442	0 (0.00; 0.00–0.83)
2023	1111	20 (1.80; 1.10–2.77)	356	20 (5.62; 3.47–8.54)	755	0 (0.00; 0.00–0.49)
2024	1031	39 (3.78; 2.70–5.14)	337	39 (11.57; 8.36–15.48)	694	0 (0.00; 0.00–0.53)
2025	936	48 (5.13; 3.81–6.74)	319	48 (15.05; 11.31–19.45)	617	0 (0.00; 0.00–0.60)

Note: Patient-year eligibility required the frozen phenotype to be satisfied on or before the first qualifying HF-related encounter in that year, at least one HF-related encounter in the year, observable medication data, and no recorded death, transfer, formal closure, or other administrative loss before contribution. Confidence intervals are exact Clopper–Pearson intervals.

**Table 3:** Concurrent foundational HFrEF medication classes at the standardized 2025 assessment (*n* = 936).

Therapy Measure	*n* (%)
Evidence-based beta-blocker	652 (69.7)
ARNI/ACEI/ARB	500 (53.4)
ARNI	164 (17.5)
ACE inhibitor	221 (23.6)
ARB	115 (12.3)
Mineralocorticoid receptor antagonist	210 (22.4)
SGLT2 inhibitor	48 (5.1)
0 concurrent classes	170 (18.2)
1 concurrent class	302 (32.3)
2 concurrent classes	308 (32.9)
3 concurrent classes	132 (14.1)
4 concurrent classes	24 (2.6)
All 4 classes—strict directly documented coverage sensitivity	14 (1.5)
All 4 classes—90-day missing-duration sensitivity	27 (2.9)

Note: Individual therapy-class rows may overlap; for the ARNI/ACEI/ARB subclassification, overlapping records were reconciled to one active renin-angiotensin-system category per patient. The 0-, 1-, 2-, 3-, and 4-class categories are mutually exclusive and exhaustive. The primary analysis used explicit supply duration when available and a 30-day active-record rule when duration was absent; the 90-day rule and strict directly documented-coverage rule were sensitivity analyses.

**Table 4:** Firth regression for recorded SGLT2 inhibitor use among patients with T2DM (*n* = 561; 64 events).

Predictor	Adjusted OR	95% CI	*p* Value
Age, per 10 years	1.20	1.02–1.43	0.028
Male sex	0.96	0.55–1.69	0.89
Nondialysis CKD	0.24	0.08–0.59	0.002
COPD	2.16	1.09–4.19	0.028
Ischemic heart disease	1.39	0.78–2.54	0.28
Index year, per year	1.76	1.45–2.18	<0.001
Observable follow-up, per year	1.11	0.96–1.29	0.16

Note: Confidence intervals were obtained by profile penalized likelihood; *p* values were obtained from penalized likelihood-ratio tests. All seven parameters were prespecified, and no stepwise selection was used. Associations are non-causal. The nondialysis-CKD estimate is exploratory because only four treated patients had nondialysis CKD.

**Table 5:** Prespecified sensitivity analyses of recorded SGLT2 inhibitor use.

Analysis	*N*	Overall Users, *n* (%; 95% CI)	T2DM, *N*	T2DM Users, *n* (%; 95% CI)	No T2DM, *N*	No T2DM Users, *n* (%; 95% CI)
Primary adult phenotype	1750	64 (3.66; 2.83–4.65)	561	64 (11.41; 8.90–14.33)	1189	0 (0.00; 0.00–0.31)
Strict two-record phenotype	1284	55 (4.28; 3.24–5.54)	438	55 (12.56; 9.60–16.03)	846	0 (0.00; 0.00–0.44)
Observable-medication cohort	1659	64 (3.86; 2.98–4.90)	532	64 (12.03; 9.39–15.10)	1127	0 (0.00; 0.00–0.33)
Complete-lookback first-record cohort	1402	50 (3.57; 2.66–4.67)	462	50 (10.82; 8.14–14.02)	940	0 (0.00; 0.00–0.39)
12-month fixed-horizon cohort	1268	27 (2.13; 1.41–3.08)	417	27 (6.47; 4.31–9.28)	851	0 (0.00; 0.00–0.43)
Measured eGFR ≥ 20, no dialysis	1406	59 (4.20; 3.21–5.38)	470	59 (12.55; 9.70–15.89)	936	0 (0.00; 0.00–0.39)
Measured eGFR ≥ 25, no dialysis	1378	58 (4.21; 3.21–5.41)	459	58 (12.64; 9.74–16.03)	919	0 (0.00; 0.00–0.40)
Broad adult HF contextual cohort	7317	707 (9.66; 8.99–10.36)	3960	707 (17.85; 16.67–19.08)	3357	0 (0.00; 0.00–0.11)

**Table 6:** Implementation barriers and actionable responses suggested by the current findings and prior Jordanian evidence.

Observed or Plausible Implementation Barrier	Actionable Response
Persistent diabetes-linked conceptualization of SGLT2i	Display SGLT2i as HFrEF therapy independent of diabetes in order sets; require an indication field; provide targeted prescriber education.
Prior authorization/formulary delay	Use standardized HFrEF authorization criteria, electronic submission, transparent renal thresholds, and rapid appeal pathways.
Renal/hemodynamic uncertainty and temporary deferral	Use protocolized review of eGFR, blood pressure, volume status, acute illness, and contraindications, with a mandatory reassessment date after temporary deferral.
Fragmented medication reconciliation and external prescribing	Implement pharmacist-led reconciliation across admission, discharge, outpatient care, and external medication sources where linkage is permitted.
No structured reason for omitted foundational therapy	Add standardized reason-for-non-use fields and dashboards reporting four-pillar completion, diabetes-stratified uptake, CKD-stratified uptake, and time-to-first-record metrics.

Note: These barriers are not established as causal mechanisms by the present retrospective cohort. The table integrates the descriptive patterns in this study with previously reported Jordanian stakeholder barriers [17] to identify testable implementation targets.

## Data Availability

Patient-level data are not publicly available because they are governed by institutional and Ministry of Health approvals. After verification against the locked analysis, the phenotype and medication dictionaries, validation matrices, reporting checklist, and analytic specification will be provided in the journal Supplementary Materials. Additional de-identified extracts remain subject to approval by the data custodian.

## Supplementary Materials

The following supporting information can be downloaded at: https://www.mdpi.com/article/10.3390/jcm15197569/s1, Table S1: Diagnosis and code dictionary; Table S2: Normalized text terms and conflict hierarchy; Table S3: Medication dictionary and active-record rules; Table S4: Independent phenotype-validation reference and results; Table S5A: Medication-classification validation matrices; Table S5B: Medication-classification performance and reviewer agreement; Table S6: Annual data-completeness and run-in audit; Table S7: Annual repeated-observation trend sensitivity analyses, 2022–2025; Table S8: Full-cohort Firth sensitivity model; Table S9: Balanced four-year cohort annual prevalence sensitivity analysis; Table S10: Time-to-first-record censoring and landmark sensitivity analyses; Table S11: Determinant-model diagnostics and time-to-event sensitivity analysis; Table S12: 2025 GDMT documentation and renin-angiotensin-system subclass sensitivity analysis.

## Abbreviations

The following abbreviations are used in this manuscript:

ACEI	Angiotensin-converting enzyme inhibitor
ARB	Angiotensin receptor blocker
ARNI	Angiotensin receptor–neprilysin inhibitor
CI	Confidence interval
CKD	Chronic kidney disease
CKD-EPI	Chronic Kidney Disease Epidemiology Collaboration
COPD	Chronic obstructive pulmonary disease
eGFR	Estimated glomerular filtration rate
EHR	Electronic health record
GEE	Generalized estimating equations
GDMT	Guideline-directed medical therapy
HF	Heart failure
HFimpEF	Heart failure with improved ejection fraction
HFmrEF	Heart failure with mildly reduced ejection fraction
HFpEF	Heart failure with preserved ejection fraction
HFrEF	Heart failure with reduced ejection fraction
ICD-10	International Classification of Diseases, Tenth Revision
IQR	Interquartile range
LVEF	Left ventricular ejection fraction
LVSD	Left ventricular systolic dysfunction
MRA	Mineralocorticoid receptor antagonist
NPV	Negative predictive value
OR	Odds ratio
PPV	Positive predictive value
RAAS	Renin-angiotensin-aldosterone system
RECORD	REporting of studies Conducted using Observational Routinely-collected health Data
SD	Standard deviation
SGLT2i	Sodium-glucose cotransporter-2 inhibitor
SMD	Standardized mean difference
T1DM	Type 1 diabetes mellitus
T2DM	Type 2 diabetes mellitus
HR	Hazard ratio
