# Supplementary material for: Recorded Adoption of SGLT2 Inhibitors from 2022 to 2025 Among Adults with an EHR-Defined HFrEF Phenotype at a Jordanian Public Hospital
Source: J Clin Med. 2026 Sep 29;15(19):7569. doi: 10.3390/jcm15197569 (PMC13666281; doi:10.3390/jcm15197569)
Supplement: Supplementary file 1 [file jcm-15-07569-s001.zip › jcm-4576418-supplementary.pdf]

Supplementary Materials

Recorded Adoption of SGLT2 Inhibitors from 2022 to 2025 Among Adults With an EHR-Defined HFrEF Phenotype at a Jordanian Public Hospital

Table S1. Diagnosis and code dictionary

Rule	Code/term family	Operational interpretation
Include with HF context	ICD-10 I50.2x	Systolic heart failure; acute, chronic, or acute-on-chronic
Conditional inclusion	ICD-10 I50.4x	Combined systolic/diastolic HF only when a reduced-EF term or LVEF ≤40% was documented
Text inclusion	HFrEF; reduced EF; systolic HF; LV systolic dysfunction	Required normalization and an HF diagnosis or problem-list entry
Insufficient alone	I50.9; heart failure; cardiomyopathy	Nonspecific terms did not establish the primary phenotype
Alternative phenotype	I50.3x; HFpEF; HFmrEF; preserved EF; mildly reduced EF	Excluded from the primary HFrEF phenotype for the relevant episode
Recovered phenotype	HFimpEF; recovered EF; improved EF with latest LVEF >40%	Later conflicting documentation superseded an older reduced-EF term
Clinical exclusion	Isolated right HF; congenital disease without LV systolic dysfunction	Excluded after manual rule hierarchy

Table S2. Normalized text terms and conflict hierarchy

Component	Examples	Rule
Normalization	Lowercase; punctuation removal; Arabic/English spelling map; abbreviation expansion	Applied before exact or boundary-aware matching
Positive terms	HFrEF; heart failure with reduced ejection fraction; systolic HF; LVSD; reduced LV function	Required HF context within the same record or problem episode
Negation	No HF; ruled out systolic dysfunction; normal LV systolic function	Negated terms were ignored as positive evidence
Historical-only rule	Remote systolic dysfunction without subsequent corroboration	Excluded from the primary cohort
Conflict priority 1	Narrative/scanned LVEF closest to index	LVEF >40% overrode an older nonspecific reduced-EF term
Conflict priority 2	Explicit cardiology/discharge phenotype	HFpEF/HFmrEF/HFimpEF overrode an older generic systolic term
Strict phenotype	Two qualifying records ≥30 days apart; one cardiology or HF discharge source	Used as a sensitivity cohort

Table S3. Medication dictionary and active-record rules

Domain	Included records	Operational rule
SGLT2i outcome	Dapagliflozin; empagliflozin	Generic/brand/formulary identifiers; cancelled, rejected, test, and historical-only entries excluded
RAAS class	Sacubitril/valsartan; ACE inhibitors; ARBs	One active class counted; overlapping agents did not create additional pillar counts
Evidence-based beta-blocker	Carvedilol; bisoprolol; metoprolol succinate	Other beta-blockers were not counted as the HFrEF foundational class
MRA	Spironolactone; eplerenone	Active order or linked issue record required
Annual activity	Explicit supply interval overlapping ≥1 day of year	If duration missing: 30 days after issue; 90-day sensitivity
2025 concurrency	Active on last HF-related encounter date in 2025	Explicit interval or issue within previous 30 days without discontinuation

Domain	Included records	Operational rule
First-record baseline	≥180 days observable medication history and no active SGLT2i	Incomplete baseline history classified as indeterminate, not incident
Strict 2025 documentation sensitivity	Foundational GDMT classes at the 2025 assessment	Require an explicitly documented supply/duration interval overlapping the assessment date; no 30- or 90-day duration imputation. ARNI, ACEI, and ARB are also reported separately after resolving overlapping RAAS records.

Table S4. Independent phenotype-validation reference and results

The final algorithm was frozen after a separate 250-record development set. The independent validation set was an unstratified simple random sample of 700 adults from the broad-HF cohort. Reviewers were blinded to algorithm status. Reference-positive documented HFrEF required narrative/scanned LVEF ≤40% or repeated explicit cardiology/discharge documentation without a later conflicting phenotype.

Reference standard	Algorithm positive	Algorithm negative	Total
Documented HFrEF	159	14	173
No documented HFrEF	8	519	527
Total	167	533	700

Metric	Estimate	Exact 95% CI
Sensitivity	91.91%	86.79–95.51
Specificity	98.48%	97.03–99.34
Positive predictive value	95.21%	90.78–97.91
Negative predictive value	97.37%	95.63–98.56
Inter-reviewer agreement before adjudication	κ=0.90	0.86–0.94

Table S5A. Medication-classification validation matrices

Medication algorithm	Validation, n	True positive	False positive	False negative	True negative
Dapagliflozin/empagliflozin	1,750	63	1	1	1,685
Evidence-based beta-blocker	300	202	4	8	86
ARNI/ACEI/ARB	300	191	5	9	95
MRA	300	80	4	5	211

Table S5B. Medication-classification performance and reviewer agreement

Medication algorithm	Sensitivity % (95% CI)	Specificity % (95% CI)	PPV % (95% CI)	NPV % (95% CI)	Pre-adjudication agreement
Dapagliflozin/empagliflozin	98.44 (91.60–99.96)	99.94 (99.67–100.00)	98.44 (91.60–99.96)	99.94 (99.67–100.00)	1,744/1,750 (99.66%); κ=0.95
Evidence-based beta-blocker	96.2 (92.6–98.3)	95.6 (89.0–98.8)	98.1 (95.1–99.5)	91.5 (83.9–96.3)	294/300 (98.0%); κ=0.95
ARNI/ACEI/ARB	95.5 (91.6–97.9)	95.0 (88.7–98.4)	97.4 (94.1–99.2)	91.3 (84.2–96.0)	292/300 (97.3%); κ=0.94
MRA	94.1 (86.8–98.1)	98.1 (95.3–99.5)	95.2 (88.3–98.7)	97.7 (94.7–99.2)	296/300 (98.7%); κ=0.96

Two clinical pharmacists with more than five years of clinical practice and EHR medication-review experience independently double-reviewed every record in the relevant validation set while blinded to the algorithm flag. A third senior clinical pharmacist adjudicated disagreements. PPV, positive predictive value; NPV, negative predictive value; κ, Cohen kappa.

Table S6. Annual data-completeness and run-in audit

Year	Status	Active, N	Complete medication source, n (%)	Users, n (%; 95% CI)	Sensitivity result
2020	Run-in	9	4 (44.4)	0 (0.00; 0.00–33.63)	Not interpreted
2021	Run-in	19	9 (47.4)	0 (0.00; 0.00–17.65)	Not interpreted
2022	Comparable	647	594 (91.8)	1 (0.15; 0.00–0.86)	0.17% in observable-medication subset

Year	Status	Active, N	Complete medication source, n (%)	Users, n (%; 95% CI)	Sensitivity result
2023	Comparable	1,111	1,046 (94.1)	20 (1.80; 1.10–2.77)	1.91% in observable-medication subset
2024	Comparable	1,031	979 (95.0)	39 (3.78; 2.70–5.14)	3.98% in observable-medication subset
2025	Comparable	936	896 (95.7)	48 (5.13; 3.81–6.74)	5.36% in observable-medication subset

Completeness required medication reconciliation or an independent linked medication source. The comparable-period GEE included 3,725 patient-year observations from 1,722 unique patients.

Table S7. Annual repeated-observation trend sensitivity analyses, 2022–2025

Analysis population	Unique patients	Patient-years	OR per calendar year	95% CI	p value
All eligible patient-years	1,722	3,725	1.88	1.59–2.23	<0.001
Patients with T2DM	—	1,217	1.93	1.62–2.31	<0.001
Observable-medication restriction	—	3,514	1.86	1.56–2.21	<0.001
Observed in ≥2 comparable years	1,091	3,094	1.82	1.51–2.20	<0.001

All models used a logit-link GEE with exchangeable working correlation and robust standard errors. Eligibility for a patient-year required phenotype satisfaction on or before the first qualifying HF-related encounter in that year, at least one HF-related encounter, observable medication data, and no prior administrative loss. The non-diabetes model was not estimable because no outcome occurred in that stratum.

Table S8. Full-cohort Firth sensitivity model

Predictor	Adjusted OR	95% CI	p value
Type 2 diabetes	289.4	40.2–2082.6	<0.001
Age, per 10 years	1.19	1.02–1.40	0.031
Male sex	0.94	0.54–1.66	0.84
Nondialysis CKD	0.25	0.09–0.61	0.003
COPD	2.09	1.06–4.04	0.034
Ischemic heart disease	1.33	0.75–2.42	0.33
Index year, per year	1.71	1.41–2.11	<0.001
Observable follow-up, per year	1.09	0.94–1.27	0.25

The T2DM coefficient is a separation-sensitive concentration estimate and is not interpreted causally. COPD is exploratory because only 16 treated patients had COPD and healthcare-contact intensity was incompletely captured. All associations are interpreted as non-causal; the CKD estimate is additionally treated as sparse-data exploratory because only four treated patients had nondialysis CKD.

Table S9. Balanced four-year cohort annual prevalence sensitivity analysis

Year	Stable cohort, N	Overall users, n (%; exact 95% CI)	Stable T2DM users, n/N (%; exact 95% CI)	Stable no-T2DM users, n/N (%; exact 95% CI)
2022	468	1 (0.21; 0.01–1.18)	1/158 (0.63; 0.02–3.48)	0/310 (0.00; 0.00–1.18)
2023	468	8 (1.71; 0.74–3.34)	8/158 (5.06; 2.21–9.73)	0/310 (0.00; 0.00–1.18)
2024	468	18 (3.85; 2.30–6.01)	18/158 (11.39; 6.89–17.41)	0/310 (0.00; 0.00–1.18)
2025	468	25 (5.34; 3.49–7.78)	25/158 (15.82; 10.51–22.47)	0/310 (0.00; 0.00–1.18)

Repeated-patient GEE trend: OR 2.04 per calendar year (95% CI 1.50–2.78; p<0.001) overall and OR 2.11 (95% CI 1.53–2.91; p<0.001) in T2DM. Absolute 2022-to-2025 changes were +5.13 percentage points overall and +15.19 percentage points in T2DM. The stable cohort required the same patient to satisfy phenotype, care-engagement, medication-observability, and administrative-status criteria in every comparable year.

Table S10. Time-to-first-record censoring and landmark sensitivity analyses

Landmark/analysis	Observed first records	Crude proportion	Kaplan–Meier estimate (95% CI)
30 days	8/1,402	0.57%	0.57% (0.29–1.13)
90 days	13/1,402	0.93%	0.94% (0.55–1.61)
180 days	19/1,402	1.36%	1.38% (0.88–2.15)
365 days	27/1,402	1.93%	2.08% (1.40–3.08)
730 days	47/1,402	3.35%	3.72% (2.78–4.97)

Verified fixed 12-month horizon	27/1,268	2.13% (95% CI 1.41–3.08)	Not applicable

For the Kaplan–Meier sensitivity analysis, time zero was the HFrEF-phenotype index date. Patients were censored at the last medication-observable date, recorded death/transfer/administrative closure, or 31 December 2025, whichever came first. Because out-of-hospital death was incompletely captured, these estimates describe time to first EHR-recorded use and are not competing-risk estimates of clinical initiation. The verified fixed 12-month estimate remains the principal timing result.

Table S11. Determinant-model diagnostics and time-to-event sensitivity analysis

Diagnostic item	Result
Spearman correlation: index year vs observable follow-up	ρ = −0.61; p<0.001
Maximum variance inflation factor	2.18
Restricted cubic-spline test: age	p=0.47
Restricted cubic-spline test: observable follow-up	p=0.33
Case-deletion influence	Maximum absolute DFBETA 0.38; no single case changed coefficient direction
Global proportional-hazards test in Cox sensitivity model	p=0.68

Exploratory Cox proportional-hazards sensitivity model in the complete-lookback T2DM cohort (n=462; 50 first-record events)

Predictor	Hazard ratio	95% CI	p value
Age, per 10 years	1.17	0.99–1.39	0.064
Male sex	0.95	0.53–1.70	0.86
Nondialysis CKD	0.32	0.11–0.80	0.016
COPD	1.71	0.85–3.44	0.13
Ischemic heart disease	1.31	0.72–2.38	0.37
Index year, per year	1.52	1.18–1.95	0.001

The Cox sensitivity model handles observable follow-up through the time-to-event framework and therefore does not include observable follow-up duration as a predictor. Associations are exploratory and non-causal. The CKD estimate remains based on sparse treated events and should not be interpreted as evidence of an independent renal effect.

Table S12. 2025 GDMT documentation and renin-angiotensin-system subclass sensitivity analysis

Therapy measure	Primary 30-day rule, n (%)	Strict directly documented coverage, n (%)
Evidence-based beta-blocker	652 (69.7)	518 (55.3)
ARNI	164 (17.5)	130 (13.9)
ACE inhibitor	221 (23.6)	173 (18.5)
ARB	115 (12.3)	86 (9.2)
ARNI/ACEI/ARB combined	500 (53.4)	389 (41.6)
MRA	210 (22.4)	149 (15.9)
SGLT2 inhibitor	48 (5.1)	36 (3.8)
All four foundational classes	24 (2.6)	14 (1.5)
All four classes—90-day sensitivity	27 (2.9)	Not applicable

Among the 1,410 positive patient-class classifications contributing to the primary 2025 analysis, 1,131 (80.2%) had an explicit duration/supply interval and 279 (19.8%) as relied on the 30-day missing-duration rule. ARNI, ACEI, and ARB categories are mutually exclusive after reconciliation. Reasons for non-use were not systematically captured and therefore were not retrospectively inferred.

.
